# Lymphatic Function in Autoimmune Diseases

**DOI:** 10.3389/fimmu.2019.00519

**Published:** 2019-03-20

**Authors:** Noa Schwartz, Madhavi Latha S. Chalasani, Thomas M. Li, Zhonghui Feng, William D. Shipman, Theresa T. Lu

**Affiliations:** ^1^HSS Research Institute, Hospital for Special Surgery, New York, NY, United States; ^2^Division of Rheumatology, Department of Medicine, Hospital for Special Surgery, New York, NY, United States; ^3^Weill Cornell Tri-Institutional MD-PhD Program, New York, NY, United States; ^4^Division of Pediatric Rheumatology, Department of Medicine, Hospital for Special Surgery, New York, NY, United States; ^5^Department of Microbiology and Immunology, Weill Cornell Medicine, New York, NY, United States

**Keywords:** lymphatics, autoimmune disease, rheumatoid arthritis, scleroderma, systemic lupus erythematosus, lymphatic massage

## Abstract

Lymphatic vessels are critical for clearing fluid and inflammatory cells from inflamed tissues and also have roles in immune tolerance. Given the functional association of the lymphatics with the immune system, lymphatic dysfunction may contribute to the pathophysiology of rheumatic autoimmune diseases. Here we review the current understanding of the role of lymphatics in the autoimmune diseases rheumatoid arthritis, scleroderma, lupus, and dermatomyositis and consider the possibility that manual therapies such as massage and acupuncture may be useful in improving lymphatic function in autoimmune diseases.

## Introduction

As early as the Fourth century, Aristotle described lymphatic vessels as fibers positioned between blood vessels and nerves, containing colorless liquid (1). While our understanding of the lymphatic system has advanced since Aristotle's time, the functional significance of the lymphatic network to health and disease is still being unraveled. The lymphatic system is a network of vessels that drains protein-rich lymph from the extracellular fluid, transports it through a series of lymph nodes (LNs), and finally returns it to the bloodstream. Beyond their role in maintaining tissue fluid homeostasis, lymphatic vessels are an important part of the immune system: they allow transport of antigens from the periphery to LNs, where immune cells are primed, expanded, and eventually transported to the site of inflammation (2, 3). In addition to ferrying lymph and immune cells, the lymphatic system itself is directly involved in immune modulation and induction of tolerance to self-antigens (4, 5). Given the function of the lymphatic system in immunity, lymphatic dysfunction may also contribute to the pathophysiology of autoimmune diseases. Here, we review the current understanding of the lymphatic function within autoimmune disease. We begin with a brief overview of the lymphatic system, discuss what is known about lymphatic function in a number of rheumatic diseases, starting from the best studied to the least studied in terms of lymphatic function, and conclude with a consideration of manual therapies as potential approaches to improve lymphatic function in disease. Our goal is to bring more attention to this under-explored, yet promising, area of study.

## Overview of the Lymphatic System

Lymphatic vessels form an extensive network throughout the body with the exception of only a few tissues including bone, heart myocardium and skeletal muscles, as well as the parenchyma of kidney, liver, and adrenal and thyroid glands. These exceptions either have little interstitial fluid or have an alternative drainage system, such as fenestrated blood vessels (6).

The lymphatic system is composed of initial lymphatic capillaries that merge to form collecting lymphatic vessels. The collecting vessels transport the lymph to and from a series of LNs and eventually drain into the thoracic duct that connects to the blood circulation by draining into the subclavian veins (6). Lymphatic capillaries are blind-ended and are composed of a thin layer of lymphatic endothelial cells (LECs) with discontinuous basement membrane and “button-like” cell junctions, allowing unidirectional flow of cells and fluid into the vessel (7). In contrast to the capillaries, collecting lymphatic vessels (LVs) have intraluminal valves that prevent backflow of lymphatic fluid (8) and several perivascular layers of lymphatic muscle cells, with characteristics of both smooth muscle cells and cardiac striated muscle cells, that provide vascular tone and rhythmic contractions of the vessels, enabling anti-gravity, active fluid transportation (9, 10). Previously, distinguishing blood endothelial cells from LECs was difficult. But recent research identified several LEC-specific markers, including Lyve-1, vascular endothelial growth factor receptor-3 (VEGFR-3), podoplanin (PDPN), and Prox-1, among others, that enabled tremendous advancements in the study of lymphatic vessel development and function (11, 12).

Previous studies have shown that the systemic vasculature can reabsorb up to 90% of extravasated water, while the remaining 10% is absorbed by the lymphatic vessels (13). However, congenital or acquired dysfunction of the lymphatic system result not only in lymphedema and its sequalae such as skin thickening, fibrosis, and adipose degeneration, but also in poor immune function, susceptibility to infections, and impaired wound healing, among other deleterious health effects (8). These observations point at the greater role lymphatic vessels have than simple fluid transportation. Literature has shown that LECs directly affect immune cell activity in many different ways, including secretion of transforming growth factor-β (TGFβ) leading to suppression of dendritic cell (DC) maturation (14); production of IL-7 to increase IL-2 sensitivity in regulatory T cells to increase their immune-regulatory function (15) and to sustain inflammation-induced lymphoid follicles in disease (16); secretion of colony stimulating factor-1 (CSF-1) promoting differentiation, proliferation and survival of macrophages that contribute to tumor growth (17). LECs also present peripheral tissue antigens together with programmed death-ligand-1 (PD-L1) leading to CD8+ T-cell response inhibition (18) and modulate CD4+ T-cell response via low level antigen presentation with MHC-II during inflammation (19). That lymphatic dysfunction exacerbates autoimmune disease is supported by the development of autoantibodies in mice lacking dermal lymphatics (5). Understanding the lymphatic system in the context of autoimmune diseases has the potential to provide insight into disease mechanisms and new approaches to treatment.

## Lymphatics and Rheumatoid Arthritis

Rheumatoid arthritis (RA) is one of the most studied autoimmune conditions, with regards to the role of lymphatics in the context of disease. RA is an autoimmune systemic disease, affecting 0.5–1% of the population, with its hallmark being symmetric polyarthritis, usually with small-joint distribution (20). Local lymph node enlargement was first described in RA in 1896 (21), but it wasn't until more specific markers of the lymphatic system were discovered that its role could be specifically investigated.

It is thought that together with the joint inflammation occurring in RA, the local lymphatics undergo two stages of alterations. As a response to the initial, pre-arthritic, synovial inflammation, the lymphatics undergo an “expansion phase,” whereby they increase their capacity to remove excess cellular debris and inflammatory cells from the site of inflammation; whether by lymphangiogenesis (22), or by increased lymphatic vessel contraction frequency (23). This process is important to allow for the resolution of the inflammatory process; if the expansion process is stunted, by inhibition of lymphangiogenesis for example, the joint inflammation becomes more severe and clinical synovitis develops (24). Beyond the lymphatic vessel changes, the draining lymph nodes themselves increase in size during the expansion phase (23, 25), likely due to increased volume and pressure of fluid within the afferent vessels (25), intra-nodal lymphangiogenesis (23), and infiltration of a unique subtype of IgM^+^CD23^+^CD21^hi^CD1d^hi^ B cells found in inflamed lymph nodes, known as Bin cells (26, 27). Nevertheless, while the removal of the excess debris is important to allow for inflammation resolution in the acute setting, the inflammatory cells, and catabolic factors that are being removed have been shown to directly damage the LECs and lymphatic muscle cells, both in the afferent lymphatic vessels and the draining lymph nodes (28). As a result of this ongoing stress on the lymphatic system, the lymphatics progress to the “collapsed phase,” in which the local lymphatic conduit system breaks down, and the lymph node is no longer able to efficiently drain the fluid from the inflamed synovium (24, 25). The lymphatic vessels are damaged, with increased leakiness and reduced contractions, leading to poor lymphatic clearance, and stasis of the inflammatory fluid within the joint and the afferent lymphatic vessels (23, 28–31). The process is thought to be mediated by several factors, including inflammatory cytokines in the vessels triggering LEC expression of inducible nitric oxide synthase (iNOS), as well as iNOS-producing activated myeloid cells, now static within the lymphatic vessels. The increased local NO production abrogates the constitutive endothelial NOS (eNOS) activity that is an important mediator of lymphatic vessel contraction (32, 33). Reduced vessel contraction is likely also due to increased fluid flow and pressure inside the vessels, beyond the vessels' ability to compensate (34, 35). At the same time, Bin cells in the draining lymph node migrate from the lymph node follicles to the sinuses, as extensively reviewed by Bouta et al. (31), leading to clogging of lymph node sinuses and blocking passive lymphatic drainage. The resulting impairment in lymphatic drainage contributes to increased joint inflammation and synovial hyperplasia, eventually leading to joint destruction (36). Importantly, known and effective RA treatments, such as tumor necrosis factor (TNF) inhibition and anti-CD20 therapy, have both shown to also have a beneficial effect on lymphatic flow. Inhibition of TNF has been shown to restore lymphatic vessel contractility (28), and anti-CD20 therapy, i.e., Rituximab, depletes Bin cells from the lymphatic sinuses, thereby promoting the restoration of lymphatic flow (37).

Preliminary work with indocyanine green near-infrared (ICG-NIR) fluorescence imaging has been promising in providing sensitive, real-time non-invasive means to evaluate the layout and function of the lymphatic vasculature (31, 38, 39), and the first study of lymphatic flow in RA patients with this modality is currently being performed (ClinicalTrials.gov NCT02680067). Meanwhile, the change in size of the local draining lymph nodes has been shown to reflect joint inflammatory activity, as well as response to therapy (40). Even prior to clinical lymphadenopathy, evaluation of draining lymph nodes of inflamed joints by power Doppler ultrasound (PDUS), demonstrated hypertrophy of the lymph node cortex, in addition to power Doppler signal amplification in cortical and hilar regions likely indicating increased flow. Importantly, these findings reversed with treatment (41). At the same time, low PDUS signal at baseline despite active arthritis, likely representing a collapsed lymph node, predicts poor clinical response to therapy (40). Similarly, in a pilot study using contrast enhanced MRI (CE-MRI) to monitor LN size before and after treatment with Certolizumab in RA patients, there was an inverse correlation between the extent of treatment-related pain relief and decrease in LN size. The LNs with the more notable reduction in size are the ones that are more likely to have undergone collapse, leading to inadequate inflammation resolution and reduced pain relief (42). Thus, in addition to providing an important, non-invasive means by which to monitor disease activity, response to therapy, and even predict prognosis; these findings also support the bi-phasic lymphatic response model, namely the “expansion phase” and “collapse phase,” seen in murine models of inflammatory arthritis.

## Lymphatics and Systemic Sclerosis

Scleroderma is an autoimmune connective tissue disease characterized by abnormalities in vasculature, immune function, and extracellular matrix that ultimately manifest as fibrosis of the cutaneous, vascular, musculoskeletal, gastrointestinal, pulmonary, cardiac, and renal systems. Although the etiology of the disease is poorly understood, vascular injury and abnormal endothelial cell function are hypothesized to be among the primary defects responsible for disease pathogenesis (43, 44).

Studies into vascular abnormalities leading to fibrosis have generally been more focused on blood endothelial cells (BECs), while the roles of LECs and lymphatic dysfunction have been less well-studied. In 1999, Leu et al. first demonstrated using lymphangiography that scleroderma lesional skin had signs of lymphatic microangiopathy, with absence or fragmentation of visualizable lymphatic networks and evidence of vessel leakiness and backflow (45). Similar findings using immunohistochemical staining of scleroderma skin recently showed a decrease in lymphatic vessels and increased cross-sectional area of the remaining vessels, suggesting vessel dilatation and a block in lymphatic flow downstream (44). The changes were most significant in the reticular dermis, with a similar trend found in the papillary dermis. Lymphatic changes have been found in other fibrosing conditions as well (Table 1), further supporting the idea that lymphatic dysfunction may be a therapeutic target in scleroderma.

**Table 1:** Summary of lymphatic dysfunction in fibrosis of different organs.

**Disease model**	**Findings**	**Mechanism/molecules involved**	**References**
**LUNG FIBROSIS**			
Human lung	Enlarged mediastinal lymph nodes (32% in SSc vs. 2% controls)		(46)
	•Increased alveolar lymphangiogenesis early in the disease •Lymphatic area directly proportional to the severity of the disease	CD11b+ macrophages form LECs in alveolar spaces of IPF patients but not controls	(47)
Radiation-exposed mouse lung	Progressive loss of pulmonary lymphatic vessels	Increase in VEGF-C and D expressing alveolar macrophages	(48)
**SKIN FIBROSIS**			
Human Skin	•Decreased lymphatic vessel counts in SSc patients •Inverse correlation between low vessel counts with fingertip ulcers		(49)
	Decreased density of reticular dermis lymphatic vessels		(44)
	Lymphatic microangiopathy		(45, 50)
Mouse tail skin radiation-induced fibrosis	Decrease in dermal capillary lymphatic vessels and LEC	TGF-β signaling inhibition protects from radiation-induced soft tissue fibrosis and lymphatic dysfunction.	(51)
**LIVER FIBROSIS**			
Sprague Dawley rat model	Increased lymphatic diameter in CCl4 induced fibrosis mice compared to control mice		(52)
Human liver	•Increase in area of each lymphatic vessel •Increase in number of lymphatic vessels per section and directly proportional to the fibrosis severity		(53)
**RENAL AND PERITONEAL FIBROSIS**			
Human kidney	•Presence of LEC in the tubulointerstitial fibrotic lesions and not in control sample •Lymphatic vessel proliferation in tubulointerstitial fibrosis and inflammatory interstitial areas, filled with mononuclear cells in the lymphatic lumen		(54)
Unilateral Ureteral Obstruction rat model	Increased lymphangiogenesis	Increased TGF-β and VEGF-C expression	(55)
Rat remnant kidney model	•Massive proliferation of lymphatic vessels in fibrotic tubulointerstitial regions. •Mononuclear clusters in lymphatic vessels		(56)

*SSc, systemic sclerosis; LEC, lymphatic endothelial cells; IPF, idiopathic pulmonary fibrosis; VEGF, vascular endothelial growth factor; TGF-β, transforming growth factor-beta*.

## Lymphatics and Systemic Lupus Erythematosus and Dermatomyositis

Systemic lupus erythematosus (SLE) is the prototypical systemic autoimmune disease, affecting between in 6.5 and 187 per 100,000 people worldwide, with a 9:1 female predominance and mortality rate that is three times that of the general population (57, 58). There have been no systematic studies of lymphatic function in SLE to date. There are, however, hints that there might be lymphatic dysfunction in SLE, as there are case reports of chylous ascites or pleural effusions, lymph fluid found in the abdomen or thoracic cavity, respectively, that can result from lymphatic obstruction in the mesentery (59–62). Lymphedema from peripheral lymphatic obstruction has also been described (63). These occurrences are rare, however, and whether more subtle problems with lymphatic flow that could perhaps result from the lymphadenopathy commonly found in SLE (64, 65) are not known.

Similarly, lymphatic function in dermatomyositis, a group of autoimmune diseases primarily directed against the muscle and skin (66), has not been systematically studied. Dermatomyositis patients can rarely present with generalized edema, which may reflect poor lymphatic function and lymphedema (67). Gottron's papules are characteristic red, raised lesions on the knuckles of dermatomyositis patients, and one study examining the histopathology of these papules noted dilated PDPN^+^ lymphatic vessels (68). Interestingly, benign lymphadenopathy is less frequent in dermatomyositis than in SLE or RA, and, because of the association of cancer with dermatomyositis in adults, lymphadenopathy in dermatomyositis has to be evaluated carefully for metastatic cancer or lymphomas (69).

## Potential Approaches to Improving Lymphatic Function in Rheumatic Diseases: New and Ancient

As we begin to understand the role of lymphatic dysfunction in autoimmune diseases, it is also time to consider how we might improve lymphatic function as part of disease treatment. Schwarz and colleagues have recently outlined molecularly targeted therapies that are currently being investigated (31). In contrast to pharmacologic approaches, manual therapies have been used since ancient times and are currently the mainstay in improving lymphatic flow in diseases. Below, we briefly discuss some of these approaches to consider their potential utility in improving lymphatic function in autoimmune diseases.

Lymphatic-directed massage techniques are used in the treatment of primary and secondary forms of lymphedema, such as that which occurs in the arms of 20% of breast cancer surgery patients when axillary lymph nodes have been removed (70). Manual lymphatic drainage (MLD) is a specific light pressure massage technique that moves from the trunk to the distal portion of the affected limb to stimulate lymph flow away from the peripheral tissue (71). Indeed, hand edema is observed in systemic sclerosis patients in the early edematous phase, and MLD has been shown to significantly reduce the swelling and improve hand function in these patients (72). Similarly, a dry brushing massage technique used in Ayurvedic medicine that originated in India 5,000 years ago is meant to relieve lymphatic congestion that is thought to contribute to stress and disease. It has also been used to reduce lymphedema and inflammation from lymphatic filariasis (73). Interestingly, the sports industry, which has been interested in promoting post-training recovery and reducing edema, is investigating the utility of peristaltic pulse dynamic compression (PPDC) devices that simulate manual lymphatic therapies. Recently, PPDC was shown to increase the pressure-to-pain threshold in elite athletes (74) and also to induce expression of anti-inflammatory genes (75), although it is yet unclear whether these effects are attributable to improving lymphatic flow. Potentially, then, lymphatic massage techniques could be used to improve lymphatic function to help reduce tissue inflammation in autoimmune diseases.

Interestingly, acupuncture, a component of traditional Chinese medicine, may potentially have a lymphatic basis. Acupoints are specific points on the body that practitioners target in an attempt to mobilize stagnant *qi*, thought to be a form of energy that flows through the meridian system and enhance well-being (76). Recent analysis of acupoints demonstrates that they co-localize with tissue planes rich in nerves, blood and lymphatic vessels, and mast cells. Acupuncture involves insertion of metal needles into the skin and spinning the needle between the acupuncturist's fingers. It has been proposed that this process disturbs local tissues and transmits a biomechanical signal to surrounding cells and structures (77) that can stimulate lymphatic vessels. Additionally, activation of the nerves or mast cells in the area could result in release of vasoactive cytokines that can then stimulate lymphatic vessels to better mobilize fluid and inflammatory cells from the area (77–80). There are observational trials showing efficacy of acupuncture on breast cancer-associated lymphedema, supporting the idea that acupuncture can modulate lymphatic function (81–83). A recent randomized controlled trial examining the ability of acupuncture to further reduce lymphedema on top of current standard therapies such as lymphatic massage drainage and compression sleeves did not show additional benefits (84). However, whether acupuncture alone is at least as good as current standard therapies is not yet known. It should be of interest to better study whether acupuncture could modulate lymphatic function to aid in the treatment of autoimmune diseases.

While the objective of these manual therapies in lymphedema is to improve lymphatic flow and reduce inflammation and swelling in the affected tissues, improving lymphatic flow has the potential to also modulate immune cell activity in a number of ways. First, as mentioned in section Overview of the Lymphatic System, LECs can directly regulate immune cell function, and stimulation of lymphatic flow can modulate the ability of LECs to regulate immune cells (85). Second, lymphatic flow, by means of transporting antigen from the periphery, can impact the tolerance and activation of lymph node lymphocytes (5). Third, cytokines expressed in peripheral tissues can impact immune function in lymph nodes (86), potentially in part by lymphatic transport to the lymph nodes (87). Here, it is possible that cytokines transported to the draining nodes can both activate and regulate lymph node responses, suggesting that improving lymphatic flow can help reduce the duration and/or magnitude of ongoing autoimmune responses. Thus, in the study mentioned above examining the effects of MLD on hand edema in scleroderma patients (72), it would be interesting to understand whether MLD reduced autoantibody levels when edema was reduced. Manual therapies to improve lymphatic flow, then, may be a well-tolerated, relatively low-cost method to improve many facets of lymphatic function to reduce inflammation and autoimmunity in rheumatic diseases.

## Conclusions and Future Directions

The lymphatic system has not been well-studied in autoimmune diseases generally, but the existing evidence, especially in RA and, to a more limited extent, in systemic sclerosis suggests that there is at least dysfunction of lymphatic flow. Further studies focused on the consequences of dysfunctional flow as well as alterations in the direct effects of lymphatic vessels and LECs on innate and adaptive immune cells should provide insights into how best to target the lymphatics in autoimmune rheumatic diseases. Additionally, understanding the causes of lymphatic dysfunction in these diseases may help us better target upstream mediators and perhaps reveal that lymphatic targeting is a mechanism of action of some medications. Finally, as we consider new approaches to targeting lymphatics in autoimmune diseases, there may be value in better understanding older approaches in the context of Twenty-First century biomedical understanding of lymphatic and immune function to expand our therapeutic armamentarium for autoimmune diseases.

## Author Contributions

All authors listed have made a substantial, direct and intellectual contribution to the work, and approved it for publication.

### Conflict of Interest Statement

The authors declare that the research was conducted in the absence of any commercial or financial relationships that could be construed as a potential conflict of interest.
